# Birth Volume and Geographic Distribution of US Hospitals With Obstetric Services From 2010 to 2018

**DOI:** 10.1001/jamanetworkopen.2021.25373

**Published:** 2021-10-08

**Authors:** Sara C. Handley, Molly Passarella, Heidi M. Herrick, Julia D. Interrante, Scott A. Lorch, Katy B. Kozhimannil, Ciaran S. Phibbs, Elizabeth E. Foglia

**Affiliations:** 1 Roberts Center for Pediatric Research, Children’s Hospital of Philadelphia, Philadelphia, Pennsylvania; 2 Leonard Davis Institute of Health Economics, The University of Pennsylvania, Philadelphia; 3 Division of Health Policy & Management, University of Minnesota School of Public Health, Minneapolis; 4 Veterans Affairs Palo Alto Health Care System, Palo Alto, California; 5 Stanford University School of Medicine, Stanford, California

## Abstract

**Importance:**

Timely access to clinically appropriate obstetric services is critical to the provision of high-quality perinatal care.

**Objective:**

To examine the geographic distribution, proximity, and urban adjacency of US obstetric hospitals by annual birth volume.

**Design, Setting, and Participants:**

This retrospective population-based cohort study identified US hospitals with obstetric services using the American Hospital Association (AHA) Annual Survey of Hospitals and Centers for Medicare & Medicaid provider of services data from 2010 to 2018. Obstetric hospitals with 10 or more births per year were included in the study. Data analysis was performed from November 6, 2020, to April 5, 2021.

**Exposure:**

Hospital birth volume, defined by annual birth volume categories of 10 to 500, 501 to 1000, 1001 to 2000, and more than 2000 births.

**Main Outcomes and Measures:**

Outcomes assessed by birth volume category were percentage of births (from annual AHA data), number of hospitals, geographic distribution of hospitals among states, proximity between obstetric hospitals, and urban adjacency defined by urban influence codes, which classify counties by population size and adjacency to a metropolitan area.

**Results:**

The study included 26 900 hospital-years of data from 3207 distinct US hospitals with obstetric services, reflecting 34 054 951 associated births. Most infants (19 327 487 [56.8%]) were born in hospitals with more than 2000 births/y, and 2 528 259 (7.4%) were born in low-volume (10-500 births/y) hospitals. More than one-third of obstetric hospitals (37.4%; 10 064 hospital-years) were low volume. A total of 46 states had obstetric hospitals in all volume categories. Among low-volume hospitals, 18.9% (1904 hospital-years) were not within 30 miles of any other obstetric hospital and 23.9% (2400 hospital-years) were within 30 miles of a hospital with more than 2000 deliveries/y. Isolated hospitals (those without another obstetric hospital within 30 miles) were more frequently low volume, with 58.4% (1112 hospital-years) located in noncore rural areas.

**Conclusions and Relevance:**

In this cohort study, marked variations were found in birth volume, geographic distribution, proximity, and urban adjacency among US obstetric hospitals from 2010 to 2018. The findings related to geographic isolation and rural-urban distribution of low-volume obstetric hospitals suggest the need to balance proximity with volume to optimize effective referral and access to high-quality perinatal care.

## Key Points

**Question:**

What were the birth volumes and geographic distributions of US obstetric hospitals from 2010 to 2018?

**Findings:**

In this cohort study of 34 054 951 births at 3207 obstetric hospitals identified using American Hospital Association data from 2010 to 2018, 56.8% of infants were born in high-volume obstetric hospitals, and 37.4% of hospitals were low volume. Among low-volume hospitals, 18.9% were isolated and 58.4% of these were rural.

**Meaning:**

In this cohort study, birth volumes varied among US obstetric hospitals, with many isolated, low-volume obstetric hospitals located in rural areas, suggesting a need to ensure access to perinatal care.

## Teaser

This cohort study examines the geographic distribution, proximity, and urban adjacency of US obstetric hospitals by annual birth volume from 2010 to 2018.

## Introduction

Approximately 4 million infants are born in the US each year^1^; patients requiring obstetric care and their infants account for 20% of hospitalizations annually.^2^ Physical access to obstetric hospitals is critical to the provision of perinatal care and is associated with an increased rate of in-hospital births and decreased rates of preterm birth, neonatal mortality, and perinatal mortality.^3,4,5^ However, the availability of US obstetric hospitals with respect to birth volume, geographic distribution among states, proximity of obstetric hospitals, and urban adjacency is not well understood. Such knowledge is fundamental to inform clinical and policy efforts to optimize perinatal regionalization, care delivery, and outcomes.

Annual obstetric hospital volume, defined as the number of births per year, is an active area of perinatal research given the positive associations between volume and outcome reported in other medical disciplines.^6,7,8^ In perinatal care, delivery at higher-volume obstetric hospitals is associated with improved infant^9,10,11,12,13^ and maternal^5^ outcomes among low-risk term infants and in high-risk populations, such as infants with very low birth weight.^14,15,16^ However, among rural hospitals, associations between the quality of obstetric care (eg, cesarean delivery without indication, labor induction without indication, and episiotomy) and volume have varied.^17^ Given the documented associations between volume and outcome in a variety of perinatal populations, high rates of adverse perinatal outcomes in the US compared with other countries,^18,19^ and marked variation in maternal^20^ and neonatal^21,22,23^ care quality and outcomes among obstetric hospitals, a comprehensive, descriptive understanding of all births and obstetric hospitals with respect to annual birth volume is crucial for advancing perinatal care. The study objective was to examine the geographic distribution, proximity, and urban adjacency of US obstetric hospitals by annual birth volume from 2010 to 2018.

## Methods

### Data and Study Population

The retrospective cohort study included hospitals with American Hospital Association (AHA) Annual Survey of Hospitals data^24^ in any year between 2010 and 2018. This study, using publicly available data, was reviewed by the Children’s Hospital of Philadelphia Research Institute Institutional Review Board, which determined that it did not meet the criteria for human participants research according to 45 CFR 102(f) and did not require informed consent. Data analysis was performed from November 6, 2020, to April 5, 2021. This study followed the Strengthening the Reporting of Observational Studies in Epidemiology (STROBE) reporting guideline.

Obstetric hospitals were identified from AHA survey data and Centers for Medicare & Medicaid Services provider of services (POS) data using previously described methods.^4,25,26^ With use of the AHA survey data, hospitals meeting all 4 of the following criteria were identified as obstetric hospitals: (1) provision of obstetric services; (2) level 1 or higher maternity care, with AHA level 1 care defined as a hospital providing services to mothers and newborns during uncomplicated deliveries; (3) at least 1 dedicated obstetric bed; and (4) 10 or more births per year. For hospitals with discrepancies in these criteria, inclusion was determined after validation of the AHA criteria against the POS data. Manual verification was performed for any remaining discrepancies or uncertainties.^27,28^ Obstetric hospitals were identified annually (hospital-year) to include those that gained or lost obstetric services during the study period.^4^

### Study Measures and Variables

We defined birth volume categories as follows: 10 to 500, 501 to 1000, 1001 to 2000, and more than 2000 births per year. Birth volume categories are not standardly defined; these categories were consistent with previous studies^11,29,30,31^ that used a threshold of 500 or fewer births per year as the smallest volume category. To describe the characteristics of obstetric hospitals and availability of services, we assessed the following measures: (1) the percentage of births within each birth volume category (in the AHA data, annually), (2) the percentage of obstetric hospitals (as hospital-years) in each birth volume category, (3) the geographic distribution of obstetric hospitals by birth volume among states, (4) the proximity of obstetric hospitals to other obstetric hospitals with respect to birth volume category, and (5) the urban adjacency of obstetric hospitals, with specific attention to isolated obstetric hospitals (defined as obstetric hospitals without another obstetric hospital within a 30-mile radius). The measurement of proximity of obstetric hospitals was chosen as a proxy for access to care and potential for regionalization because it is easier to shift births a short distance compared with a long distance. The percentages of births and obstetric hospitals and geographic distribution were determined using AHA survey variables. Obstetric hospital proximity was defined as obstetric hospitals within a straight-line distance of 30 miles or less, and isolated obstetric hospitals were defined as those without another obstetric hospital within a 30-mile radius.^32,33,34^ Straight-line distance was used because of the study scope and associated computation time. Previous analyses have demonstrated that straight-line distance and driving distance produce similar results.^35,36,37^ When proximity of obstetric hospitals was assessed, the nearest hospital was mutually exclusive, referencing the highest-volume hospital. Urban adjacency was defined using the 12 urban adjacency influence codes (UICs) developed by the US Department of Agriculture Economic Research Service.^38^

We obtained hospital characteristics from the AHA survey data. Characteristics included median number of births per year; hospital ownership; teaching status; designation as a community hospital, rural referral center, or critical access hospital; core-base statistical area type; and county UIC descriptions.^38^ These descriptions use the US Office of Management and Budget’s standard definitions of metropolitan statistical areas, which define metropolitan as counties containing an urban core of more than 50 000 residents, micropolitan as counties with a population center of 10 000 to 50 000, and noncore as counties with no population center of 10 000 or larger.^39^ We condensed the 12 UICs into 6 categories: large metropolitan area (≥1 million residents) (UIC 1); small metropolitan area (≤1 million residents) (UIC 2); micropolitan, urban adjacent (UICs 3 and 5); micropolitan, non–urban-adjacent (UIC 8); noncore, urban-adjacent (UICs 4, 6, 7, 9, and 10); and noncore, non–urban-adjacent (UICs 11 and 12). We examined AHA variables related to infant services, including median number of bassinets, neonatal intensive care, and intermediate neonatal care. We also examined a composite indicator of neonatal services present, as indicated by availability of neonatal intensive care, availability of intermediate neonatal care, or whether the POS file indicated neonatal intensive care services were available.

### Statistical Analysis

We compared hospital characteristics and available infant services by hospital-year among birth volume categories using χ^2^ and Kruskal-Wallis tests. We examined the percentage of births and percentage of hospitals in each volume category at the state level by year. Using the mapping command grmap in Stata, version 16 (StataCorp LLC) and the most recent data from 2018, we first mapped the percentage of births in low-volume obstetric hospitals by state and then overlaid hospitals by birth volume category to assess the geographic distribution across the US. Then, using obstetric hospital latitude and longitude, we calculated the proximity of obstetric hospitals for each year by birth volume using the Haversine formula.^36,40^ This calculation was used to assess whether an obstetric hospital was in proximity (located within a straight-line distance of 30 miles) of another obstetric hospital and, if so, the highest-volume hospital within the 30-mile radius. We evaluated the number of births occurring in hospitals with and without proximity to obstetric hospitals with higher annual birth volumes. We also examined the geographic distribution and urban adjacency of isolated obstetric hospitals. Given potential differences in low-volume urban (metropolitan) and rural (nonmetropolitan) obstetric hospitals, we compared hospital characteristics between the 2 groups and assessed the geographic distribution of rural and urban low-volume obstetric hospitals across the US. A 2-sided *P* = .05 was the threshold for significance. Analyses were performed using Stata, version 16.

## Results

From 2010 through 2018, we identified 26 900 hospital-years of obstetric hospital data from 3207 distinct hospitals, capturing 34 054 951 associated births. A total of 2750 hospitals (85.8%) were included in the cohort throughout the entire study period, and hospitals contributed a mean of 8.4 years of data. All hospital characteristics and available infant services differed by birth volume category (Table 1).

**Table 1.** Hospital Characteristics and Available Infant Services by Annual Birth Volume From 2010 to 2018

Characteristic	Hospitals^a^					*P* value
	Total	10-500 births/y	501-1000 births/y	1001-2000 births/y	>2000 births/y	
Hospital-years, No.^b^	26 900	10 064	5784	5627	5425	NA
Births/y, median (IQR)	755 (326-1671)	252 (134-363)	710 (597-853)	1382 (1172-1645)	3034 (2442-4053)	<.001
Ownership or control						
For profit	4007 (14.9)	1152 (11.5)	1214 (21.0)	1050 (18.7)	591 (10.9)	<.001
Nonprofit	17 523 (65.1)	5877 (58.4)	3769 (65.2)	3737 (66.4)	4140 (76.3)	
Government	5370 (20.0)	3035 (30.2)	801 (13.9)	840 (14.9)	694 (12.8)	
Teaching status						
Nonteaching	15 268 (56.8)	8138 (80.9)	3604 (62.3)	2436 (43.3)	1090 (20.1)	<.001
Minor teaching	9645 (35.9)	1893 (18.8)	2063 (35.7)	2795 (49.7)	2894 (53.4)	
Major teaching	1987 (7.4)	33 (0.3)	117 (2.0)	396 (7.0)	1441 (26.6)	
Community hospital	26 374 (98.0)	9818 (97.6)	5612 (97.0)	5558 (98.8)	5386 (99.3)	<.001
Rural referral center	1974 (7.3)	393 (3.9)	835 (14.4)	545 (9.7)	201 (3.7)	<.001
Critical access hospital	4158 (15.5)	4060 (40.3)	96 (1.7)	0	2 (<0.1)	<.001
Core base statistical area type						
Metropolitan	17 239 (64.1)	2848 (28.3)	3800 (65.7)	5188 (92.2)	5403 (99.6)	<.001
Micropolitan	5578 (20.7)	3346 (33.3)	1784 (30.8)	426 (7.6)	22 (0.4)	
Rural	4083 (15.2)	3870 (38.5)	200 (3.5)	13 (0.2)	0	
Urban adjacency influence description^c^						
Metropolitan						<.001
Large	9522 (35.4)	1239 (12.3)	1971 (34.1)	2773 (49.3)	3539 (65.2)	
Small	7957 (29.6)	1667 (16.6)	1943 (33.6)	2479 (44.1)	1868 (34.4)	
Micropolitan						
Urban adjacent	3233 (12.0)	2103 (20.9)	947 (16.4)	181 (3.2)	2 (<0.1)	
Not urban adjacent	2125 (7.9)	1192 (11.8)	734 (12.7)	183 (3.3)	16 (0.3)	
Noncore						
Urban adjacent	1950 (7.3)	1846 (18.3)	97 (1.7)	7 (0.1)	0	
Not urban adjacent	2113 (7.9)	2017 (20.0)	92 (1.6)	4 (0.1)	0	
Available infant services						
Bassinets, median (IQR), No.	15 (8-24)	7 (4-10)	14 (10-19)	21 (16-26)	35 (26-47)	<.001
Neonatal intensive care	9820 (36.5)	619 (6.2)	1395 (24.1)	3037 (54.0)	4769 (87.9)	<.001
Neonatal intermediate care	5993 (22.3)	459 (4.6)	1090 (18.9)	1915 (34.0)	2529 (46.6)	<.001
Any neonatal intermediate or intensive care^d^	12 056 (44.8)	935 (9.3)	2127 (36.8)	3900 (69.3)	5094 (93.9)	<.001

Abbreviation: NA, not applicable.

^a^ Data are presented as number (percentage) of hospitals unless otherwise indicated.

^b^ A hospital-year was defined as 1 year of center data in the American Hospital Association Annual Survey of Hospitals^24^ (eg, if a hospital had data for all 9 years of the study, the institution would be represented by 9 hospital-years).

^c^ Metropolitan was defined as counties containing an urban core of at least 50 000 residents, micropolitan as counties with a population center of 10 000 to 50 000, and noncore as counties with no population center of 10 000 or larger based on the US Office of Management and Budget’s standard definition of metropolitan statistical areas.^39^ Counties were further classified by whether they were adjacent to urban or metropolitan areas based on urban adjacency influence codes.^38^ Large metropolitan hospitals were defined as those located in a county with a population of at least 1 million and small as those in a county with a population less than 1 million.

^d^ Any neonatal intermediate or intensive care was defined by either the designation of neonatal intermediate or intensive care in the American Hospital Association survey data or as indicated by the Centers for Medicare & Medicaid Services data provider of services file.^26^

**Figure 1.**
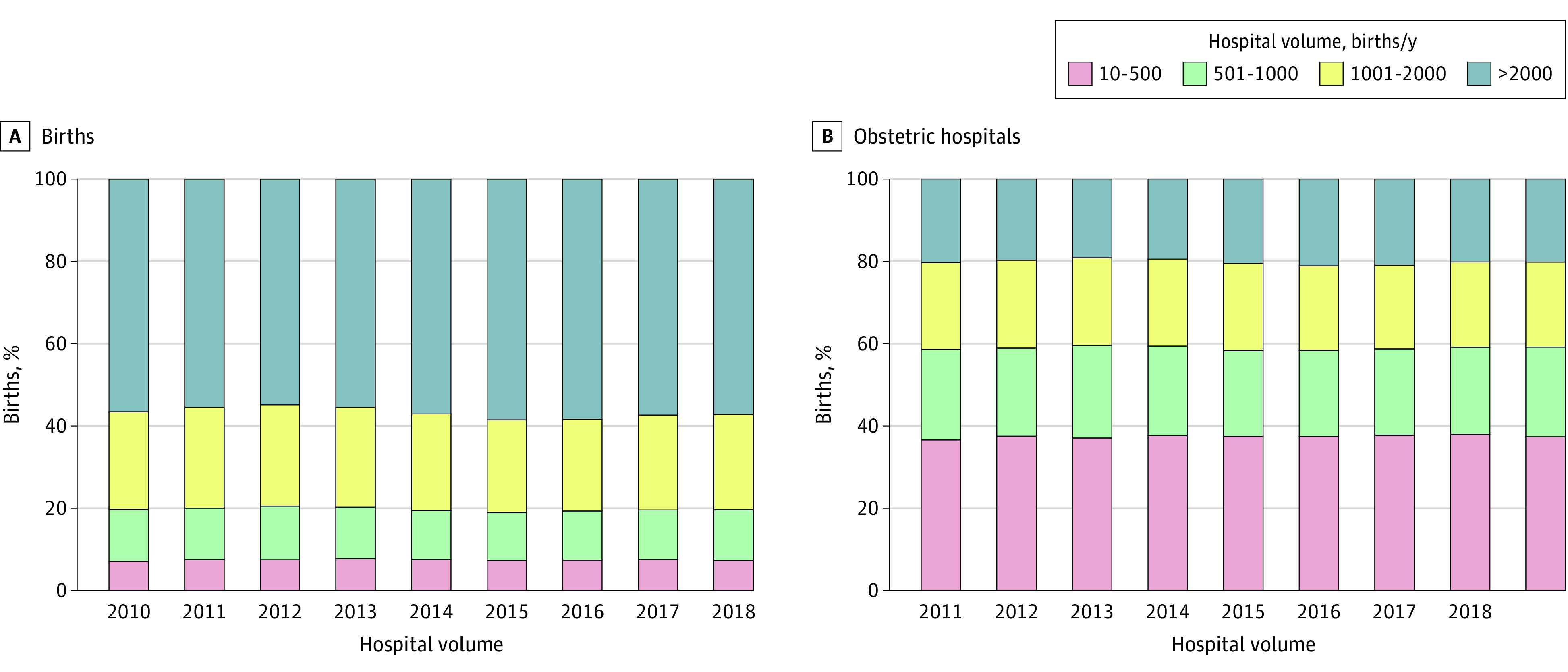
Percentage of Births and Obstetric Hospitals at the State Level by Hospital Volume Category, 2010 to 2018

Most infants (19 327 487 [56.8%]) were delivered in obstetric hospitals with more than 2000 births/y (high volume). The remaining births were distributed across volume categories: 8 002 380 (23.5%) occurred in obstetric hospitals with 1001 to 2000 births/y, 4 196 825 (12.3%) in obstetric hospitals with 501 to 1000 births/y, and 2 528 259 (7.4%) in obstetric hospitals with 10 to 500 births/y (low volume). The proportion of births by volume category was consistent during the study period (Figure 1A).

The largest group of obstetric hospitals in the US (37.4%; 10 064 hospital-years) was low volume. The relative proportion of hospital-years by birth volume category was similar across the 3 other groups, with 21.5% having 501 to 1000 births/y (5784 hospital-years), 20.9% having 1001 to 2000 births/y (5627 hospital-years), and 20.2% having 2000 or more births per year (5425 hospital-years). This distribution did not change during the study period (Figure 1B).

**Figure 2.**
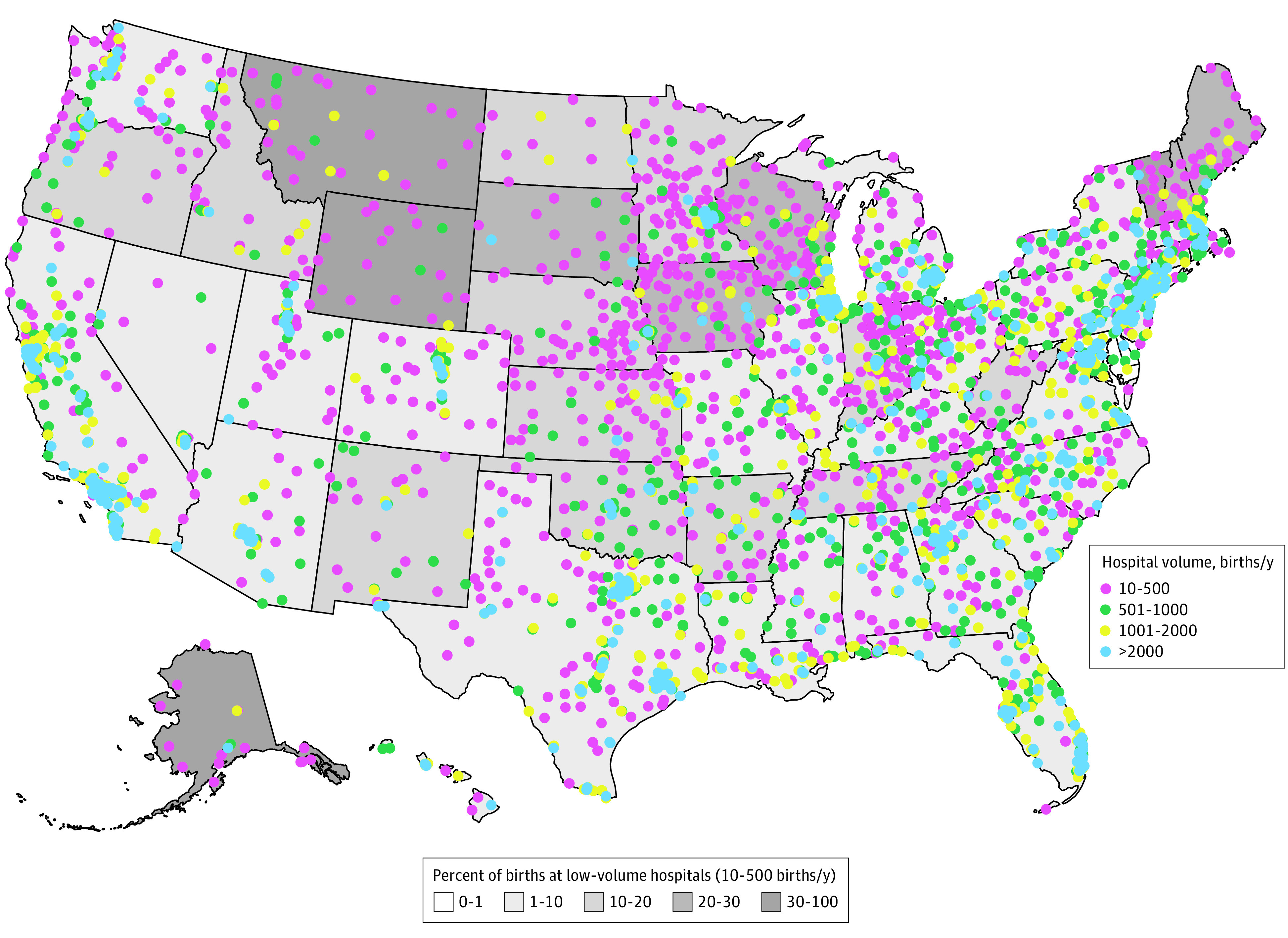
Geographic Distribution of US Obstetric Hospitals by Volume Category in 2018

In 2018, 46 states had births and obstetric hospitals in all birth volume categories (Figure 2). Exceptions included Delaware, which had no low-volume obstetric hospitals, and Maryland, in which low-volume obstetric hospitals accounted for less than 1% of births. Conversely, neither Montana nor Wyoming had obstetric hospitals with 2000 or more births per year. In 4 states (Alaska, Montana, Vermont, and Wyoming), more than 30% of births occurred in low-volume obstetric hospitals.

**Figure 3.**
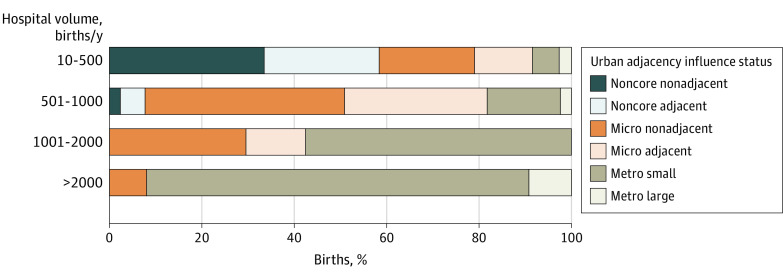
Urban Adjacency of Isolated Obstetric Hospitals From 2010 to 2018 Isolated hospitals were defined as obstetric hospitals without another obstetric hospital within a 30-mile radius. Metro indicates metropolitan; micro, micropolitan.

When proximity was assessed, 18.9% (1904 hospital-years) of low-volume hospitals were not within 30 miles of another obstetric hospital (ie, isolated), 26.5% (2665 hospital-years) were only within 30 miles of another low-volume hospital, and 23.9% (2400 hospital-years) were within 30 miles of a hospital with more than 2000 births per year (Table 2). Of the 2 528 259 infants born in a low-volume hospital, 1 215 072 (62.3%) were born in a hospital within 30 miles of a higher-volume hospital, and 952 187 (37.7%) were born in either an isolated or low-volume hospital more than 30 miles away from a higher-volume hospital. The percentage of isolated obstetric hospitals decreased with increasing birth volume category, with 18.9% (1904 hospital-years) of low-volume obstetric hospitals identified as isolated compared with 1.6% (87 hospital-years) of high-volume obstetric hospitals (Table 2). Among isolated hospitals, 58.4% (1112 hospital-years) of low-volume obstetric hospitals were located in noncore areas, and 33.5% (638 hospital-years) were located in both noncore and nonadjacent areas; whereas 7.7% (39 hospital-years) with 501 to 1000 births per year were in noncore areas, and 43.2% (218 hospital-years) were nonadjacent to a larger metropolitan area. Most isolated obstetric hospitals with 1001 to 2000 births per year (57.5% [107 hospital-years]) were in small metropolitan areas (Figure 3). Among isolated high-volume hospitals, 92.0% (80 hospital-years) were located in small or large metropolitan areas (Figure 3). The distribution of isolated hospitals across the US is shown in eFigure 1 in the Supplement. The analysis of urban and rural small-volume hospitals revealed differences in hospital characteristics and the presence of such hospitals across states (eTable and eFigure 2 in the Supplement).

**Table 2.** Proximity of US Obstetric Hospitals by Birth Volume Category From 2010 to 2018^a^

Birth volume	No.	Highest-volume category obstetric hospital within 30 miles				
		No obstetric hospital	10-500 births/y	501-1000 births/y	1001-2000 births/y	>2000 births/y
**10-500 births/y**						
Hospitals, hospital-years	10 064	1904 (18.9)	2665 (26.5)	1612 (16.0)	1483 (14.7)	2400 (23.9)
Births, No. (%)	2 528 259	412 111 (16.3)	540 076 (21.4)	413 971 (16.4)	405 594 (16.0)	755 507 (29.9)
**501-1000 births/y**						
Hospitals, hospital-years	5784	505 (8.7)	618 (10.7)	610 (10.6)	867 (15.0)	3184 (55.1)
Births, No. (%)	4 196 825	354 426 (8.5)	427 780 (10.2)	426 354 (10.2)	627 157 (14.9)	2 361 108 (56.3)
**1001-2000 births/y**						
Hospitals, hospital-years	5627	186 (3.3)	251 (4.5)	479 (8.5)	716 (12.7)	3995 (71.0)
Births, No. (%)	8 002 380	250 552 (3.1)	346 001 (4.3)	658 388 (8.2)	1 018 135 (12.7)	5 729 304 (71.6)
**>2000 births/y**						
Hospitals, hospital-years	5425	87 (1.6)	66 (1.2)	189 (3.5)	465 (8.6)	4618 (85.1)
Births, No. (%)	19 327 487	226 407 (1.2)	181 391 (0.9)	534 729 (2.8)	1 432 214 (7.4)	16 952 746 (87.7)

^a^ Proximity was defined as a straight-line distance of 30 miles or less. The nearest obstetric hospital was mutually exclusive, referencing the highest-volume hospitals within 30 miles or less.

## Discussion

This cohort study showed marked variations in birth volume, geographic distribution, proximity, and rural-urban distribution of US obstetric hospitals. These findings have potential implications for perinatal health. Although most infants in the US were born in high-volume obstetric hospitals, 2 528 259 births occurred in low-volume obstetric hospitals during the study period. Whereas 62.3% of these infants were born within 30 miles of a higher-volume obstetric hospital, the remaining 37.7% were born in either an isolated hospital or a hospital more than 30 miles from a higher-volume hospital. Overall, 18.9% of low-volume obstetric hospitals were considered isolated, and of these, 58.4% were located in rural areas. Given the importance of access to obstetric services^3,4,5^ and data supporting an association between volume and outcome in neonatal care,^9,10,11,12,14,15,16^ these findings may be particularly informative when approaches to improve perinatal care delivery and associated policies are being considered.

The findings of this study suggest the importance of low-volume obstetric hospitals in clinical and policy efforts to improve the US perinatal care system. A large number of births occurred in low-volume obstetric hospitals, and most of the obstetric hospitals had low birth volumes. In addition, low-volume obstetric hospitals were present across the rural-urban distribution, from large metropolitan areas to the most rural, noncore, non–urban-adjacent areas. This variation was also reflected in their proximity to other obstetric hospitals. A focus on low-volume facilities represents a shift in perinatal research and policy attention; both are predominantly based on evidence derived from academic health centers or teaching hospitals, which tend to be high-volume, urban facilities that provide care for high-risk infants. Regionalization research has historically focused on infants at high risk, including preterm infants (≤37 weeks’ gestation) or those with very low birth weight (<1500 g).^14,41,42,43,44,45^ Available studies of term infants or those with a birth weight greater than 2500 g suggest that infants born in hospitals with lower birth volumes are at greater risk for adverse outcomes,^9,10,11,12^ although associations in rural hospitals have varied with regard to volume.^17^ In the present study, low-volume obstetric hospitals accounted for 37.4% of obstetric hospital-years, nearly double the number in each of the other 3 birth volume categories. The volume of infant births across low-volume hospitals in the US during the study period suggests the need for further exploration of associations between birth volume and patient outcomes, particularly given the geographic variation in the proportion of low-volume hospitals among states.

The assessment of obstetric hospital proximity revealed the number of isolated obstetric hospitals, most of which were low volume and at elevated risk of closure.^27^ Although fewer isolated hospitals with higher birth volumes may be expected, the distribution of isolated obstetric hospitals across UICs warrants discussion with respect to both perinatal regionalization and rural-urban distribution of isolated hospitals. Isolated high-volume hospitals were relatively uncommon, and 92.0% were located in metropolitan areas. This metropolitan-centered population of obstetric hospitals may reflect intentional efforts to consolidate and regionalize perinatal services with the goal of improving perinatal care or unplanned closures of smaller-volume obstetric hospitals—those with 500 to 1000 births/y.^3^ Among isolated hospitals with 501 to 1000 births/y, most were serving micropolitan areas (those with centralized populations of 10 000 to 50 000). Some of these (43.2%) were nonadjacent to a larger metropolitan area, suggesting that they provide important services to the immediate and, potentially, greater surrounding area. Most isolated low-volume obstetric hospitals (58.4%) were in the most rural noncore areas. Of note, 33.5% of these hospitals were not in areas adjacent to micropolitan or metropolitan centers. Remote rural obstetric hospitals are at greatest risk of losing obstetric services, and loss of these services is associated with more out-of-hospital births, more births in hospitals without obstetric services, and more preterm births.^4^ Although Kroelinger et al^46^ found that most women of reproductive age had access to critical care obstetric services (within a straight-line distance of 50 miles), geographic disparities disproportionately affect communities in the rural US. Isolated obstetric hospitals are critical to maintaining access to obstetric services for remote, rural communities, where rates of both maternal and infant morbidity and mortality are increased.^47,48,49^

The perinatal literature on the association of volume with outcome includes assessments of a variety of thresholds to define delivery volume. Previous studies assessed birth volume by tertiles and quartiles,^29,50^ suggested differential birth volume thresholds among rural and nonrural hospitals,^30,50^ used a minimum number of deliveries ranging from 10 to 50,^4,31,50^ and classified very low-volume or low-volume obstetric hospitals as those with 200 or fewer, 500 or fewer, 650 or fewer, 1000 or fewer, or 1500 or fewer deliveries per year.^31,50,51,52^ After examining the distribution across quartiles, which did not align with those from previous studies, we defined low volume as 10 to 500 births/y to be consistent with recent literature focused on small-volume hospitals.^11,31^ If a less stringent definition, such as 1000 or fewer deliveries per year, were used in the present study, the number of US hospitals that would qualify as low-volume would increase by 1.5-fold and account for 2.5-fold more births. Variation in threshold differences in the study population and location (US-based vs international) makes direct comparison of studies difficult. However, these comparisons are necessary to inform the study of the association between volume and outcome in perinatal care.

With one-fifth of US residents living in rural counties,^53^ approaches to regionalize perinatal care should balance access, proximity, resources, and quality of care. Approaches to reorganization and regionalization have had varied effects. Although countrywide regionalization efforts in Portugal were associated with a decrease in maternal, perinatal, and neonatal mortality rates,^52^ closures of low-volume (<150 births/y) obstetric hospitals in British Columbia, Canada, had no association with outcomes^54^; obstetric unit closures in Philadelphia, Pennsylvania, were associated with increases in neonatal and perinatal mortality.^3^ The distinct geographic characteristics and markedly different health care system in the US limit applicability of external approaches to regionalization. US-based programs, such as the Rural Maternity and Obstetrics Management Strategies, provide support for rural-specific interventions regarding consolidation of services, care coordination, access to specialty care, and financial sustainability.^55^ To be effective in improving perinatal care delivery, policy and programmatic interventions should account for the US context.

The present study has potential implications. First, these findings highlight the need to include low-volume hospitals in discussions of and programs for perinatal care delivery. Second, additional studies, especially pertaining to low-volume hospitals, may help to begin to define facility and community characteristics needed to maintain safe obstetric and neonatal care, including adequate obstetric and neonatal staff with appropriate skills. Third, clinical and policy efforts toward regionalization should take into account the distances between obstetric hospitals and whether a hospital is isolated. Additional factors, including maternal and neonatal levels of care and health insurance coverage to ensure financial access to proximate hospitals, should be considered.^56,57,58,59^ US policy efforts that could be adapted based on findings from other countries include requiring representative members from rural and low-volume hospitals on decision-making bodies and councils, legislated and adequately financed perinatal regionalization programs, certificate-of-need programs, formal designation of levels of care, development of perinatal quality collaboratives, data sharing and interoperability of electronic medical records, and improved financial incentives.^60^ The potentially competing concerns of adequate volume and adequate proximity highlight the need for thoughtful discussion and policies about the optimization of perinatal care delivery.

### Limitations

This study has limitations. The self-reported nature of the AHA survey variables used to identify obstetric hospitals and volume thresholds is a limitation. To decrease potential misclassification of obstetric hospitals, we included an additional data source (POS file) and performed manual verification. In this analysis, we did not specify different volume thresholds by hospital location (rural vs urban) because we were interested in a high-level understanding of all US obstetric hospitals. Such analysis is warranted when outcomes are assessed, which was not the objective of this study. In addition, the AHA data do not include information regarding potentially relevant hospital resources (eg, types of health care professionals, available subspecialties, staffing ratios) or quality measures.

## Conclusions

This cohort study revealed marked variation in birth volume, geographic distribution, proximity, and urban adjacency of US obstetric hospitals. The large number of low-volume obstetric hospitals and the substantial number of associated births suggests the need for a better understanding of the context of low-volume hospitals in perinatal care delivery. These findings also suggest the need to balance care availability and sufficient patient volume by ensuring access and effective referral to optimize high-quality perinatal care. This work may inform ongoing discussions regarding perinatal care regionalization policy to improve maternal and infant outcomes in the US and highlights the particular concerns of isolated obstetric hospitals in these discussions.

## Notes

Supplement. **eFigure 1.** Geographic Distribution of US Isolated Obstetric Hospitals by Volume Category in 2018 **eTable.** Hospital Characteristics of Rural and Urban Low Volume Obstetric Hospitals, 2010-2018 **eFigure 2.** Geographic Distribution of US Rural and Urban Low Volume Obstetric Hospitals in 2018
