# Supplementary material for: Birth Volume and Geographic Distribution of US Hospitals With Obstetric Services From 2010 to 2018
Source: JAMA Netw Open. 2021 Oct 8;4(10):e2125373. doi: 10.1001/jamanetworkopen.2021.25373 (PMC8501399; doi:10.1001/jamanetworkopen.2021.25373)
Supplement: Supplement. — eFigure 1. Geographic Distribution of US Isolated Obstetric Hospitals by Volume Category in 2018 eTable. Hospital Characteristics of Rural and Urban Low Volume Obstetric Hospitals, 2010-2018 eFigure 2. Geographic Distribution of US Rural and Urban Low Volume Obstetric Hospitals in 2018 [file jamanetwopen-e2125373-s001.pdf]

Supplementary Online Content

Handley SC, Passarella M, Herrick HM, et al. Birth volume and geographic distribution of US hospitals with obstetric services from 2010 to 2018. *JAMA Netw Open*. 2021;4(10):e2125373. doi:10.1001/jamanetworkopen.2021.25373

eFigure 1. Geographic Distribution of US Isolated Obstetric Hospitals by Volume Category in 2018

eTable. Hospital Characteristics of Rural and Urban Low Volume Obstetric Hospitals, 2010-2018

eFigure 2. Geographic Distribution of US Rural and Urban Low Volume Obstetric Hospitals in 2018

This supplementary material has been provided by the authors to give readers additional information about their work.

© 2021 Handley SC et al. *JAMA Network Open*.

eFigure 1. Geographic distribution of US isolated obstetric hospitals by volume category in 2018

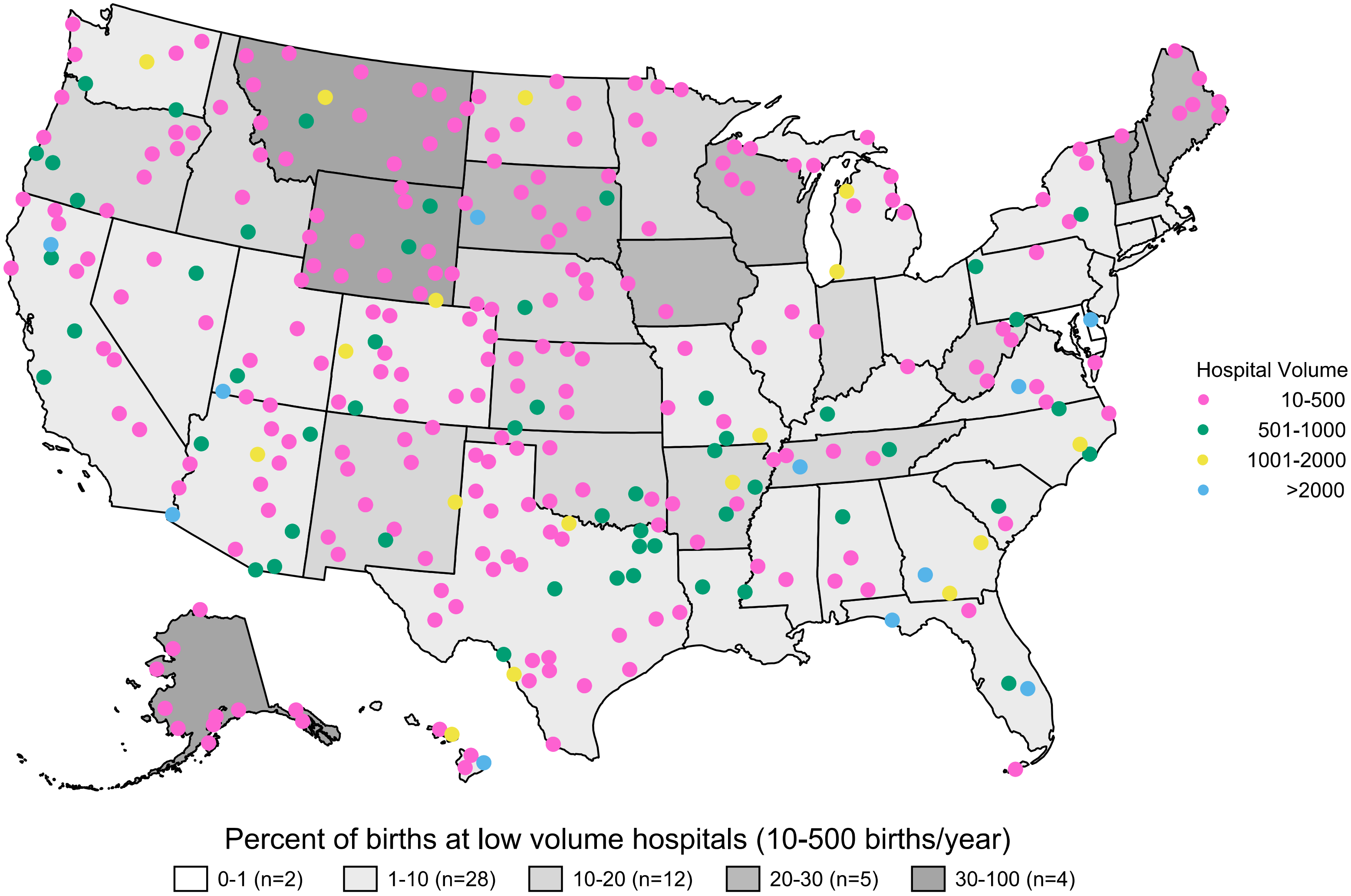

Isolated obstetric hospitals are obstetric hospitals without another obstetric hospital within a 30-mile straight-line distance.

© 2021 Handley SC et al. *JAMA Network Open*.

eTable. Hospital characteristics of rural and urban low volume obstetric hospitals, 2010-2018

	Total	Rural[a] Low Volume Obstetric Hospitals	Urban[b] Low Volume Hospitals	p-value
Hospital-years[c]	N=10,064	N=7,158	N=2,906	
Births/year, Median (IQR)	252 (134-363)	221 (118-336)	316 (203-415)	<0.001
**Hospital Characteristics**				
Ownership/Control				<0.001
For-Profit	1,152 (11.5%)	618 (8.6%)	534 (18.4%)	
Non-Profit	5,877 (58.4%)	4,149 (58.0%)	1,728 (59.5%)	
Government	3,035 (30.2%)	2,391 (33.4%)	644 (22.2%)	
Teaching Status:				<0.001
Non-Teaching	8,138 (80.9%)	6,103 (85.3%)	2,035 (70.0%)	
Minor Teaching	1,893 (18.8%)	1,055 (14.7%)	838 (28.8%)	
Major Teaching	33 (0.3%)	0 (0.0%)	33 (1.1%)	
Community Hospital	9,818 (97.6%)	7,037 (98.3%)	2,781 (95.7%)	<0.001
Rural Referral Center	393 (3.9%)	301 (4.2%)	92 (3.2%)	0.015
Critical Access Hospital	4,060 (40.3%)	3,496 (48.8%)	564 (19.4%)	<0.001
**Available Infant Services**				
Number of bassinets	7 (4-10)	6 (4-10)	9 (6-13)	<0.001
Neonatal intensive care	619 (6.2%)	327 (4.6%)	292 (10.1%)	<0.001
Neonatal intermediate care	459 (4.6%)	270 (3.8%)	189 (6.5%)	<0.001
Any neonatal intermediate/intensive care[d]	935 (9.3%)	514 (7.2%)	421 (14.5%)	<0.001

Low volume obstetric hospitals are defined has having 10-500 births/year.
[a]Rural hospitals are those identified by urban-adjacency codes 3-12. This includes micropolitan, which is defined as counties with a population center of 10,000-50,000 and noncore as counties with no population center of 10,000 or larger based on the Office of Management and Budget's standard definition of Metropolitan Statistical Areas.
[b]Urban hospitals are those identified by urban-adjacency codes 1-2, defined as metropolitan counties containing an urban core of at least 50,000 residents.
[c]Hospital-years: A hospital year is defined as one year of center data in the AHA (e.g. if a hospital had AHA data for all nine years of the study the institution would be represented by 9 hospital years).
[d]Any neonatal intermediate/intensive care was defined by either the designation of neonatal intermediate or intensive care in the AHA survey data or as indicated by the Centers for Medicare and Medicaid Services data provider of services file.

© 2021 Handley SC et al. *JAMA Network Open*.

eFigure 2. Geographic distribution of US rural and urban low volume obstetric hospitals in 2018

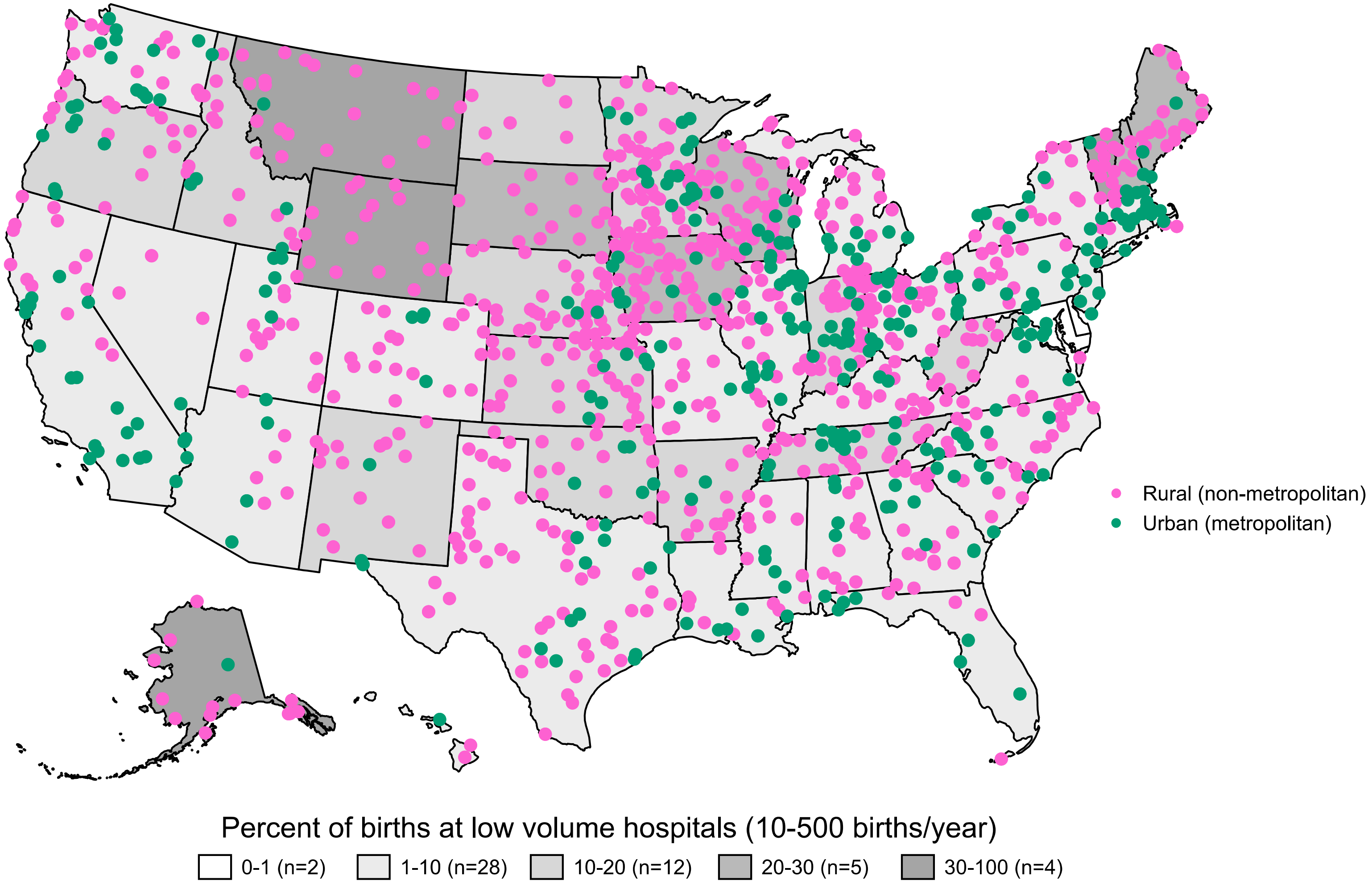

Rural hospitals are those identified by urban-adjacency codes 3-12. This includes micropolitan, which is defined as counties with a population center of 10,000-50,000 and noncore as counties with no population center of 10,000 or larger based on the Office of Management and Budget's standard definition of Metropolitan Statistical Areas.

© 2021 Handley SC et al. *JAMA Network Open*.

Urban hospitals are those identified by urban-adjacency codes 1-2, defined as metropolitan counties containing an urban core of at least 50,000 residents.

© 2021 Handley SC et al. *JAMA Network Open*.
